# Effect of dysphagia rehabilitation in patients receiving enteral nutrition at home nursing care: A retrospective cohort study

**DOI:** 10.1111/joor.13030

**Published:** 2020-07-13

**Authors:** Hiroyasu Furuya, Takeshi Kikutani, Kumi Igarashi, Keiichiro Sagawa, Yuri Yajima, Reiko Machida, Takashi Tohara, Noriaki Takahashi, Fumiyo Tamura

**Affiliations:** ^1^ Tama Oral Rehabilitation Clinic The Nippon Dental University Tokyo Japan; ^2^ Division of Rehabilitation for Speech and Swallowing Disorder The Nippon Dental University Hospital Tokyo Japan; ^3^ Division of Clinical Oral Rehabilitation The Nippon Dental University Graduate School of Life Dentistry Tokyo Japan

**Keywords:** dysphagia rehabilitation, enteral nutrition, home nursing care, oral intake, swallowing function

## Abstract

**Objective:**

We considered the effect of dysphagia rehabilitation and investigated parameters associated with the resumption of oral intake in the elderly patients receiving home nursing care who were not eating by mouth.

**Methods:**

The participants were 116 patients aged ≥65 years (66 men and 50 women, mean age 79.7 ± 8.9 years) who were receiving home nursing care and not eating by mouth because of dysphagia. All patients underwent dysphagia rehabilitation for 6 months with the objective of resuming oral intake. After 6 months of dysphagia rehabilitation, the patients’ eating status was assessed using the Functional Oral Intake Scale (FOIS) and the associations of the post‐intervention FOIS score with age, history of pneumonia, duration of enteral nutrition, body mass index (BMI), alertness, physical function (ability to walk) and swallowing function at the initial examination.

**Results:**

Functional Oral Intake Scale scores increased significantly after 6 months rather than those at the initial evaluation (*P* < .001). Eighty patients (69.0%) resumed oral intake (FOIS score ≥2), thirty patients (25.9%) of whom became capable of daily oral intake (FOIS score ≥3). Swallowing function was associated with the resumption of oral intake. In addition, physical function before dysphagia rehabilitation was an important factor to resume daily oral intake.

**Conclusions:**

The results of the present study suggest that the resumption of oral intake by patients receiving enteral nutrition requires improvement in swallowing function. In addition, anyone who cannot walk may not recover daily oral intake.

## 1 INTRODUCTION

When dysphagia is sufficiently severe to prevent oral intake, alternative nutrition methods must be used to sustain life.^1^, ^2^ Dysphagia is a common disability in elderly people, and as the population ages, the number of patients with dysphagia is predicted to increase, resulting in a corresponding increase in the number of patients requiring enteral or parenteral nutrition.

Because eating is also related to quality of life (QOL),^3^ resuming oral intake is regarded as important. However, if enteral nutrition is continued for too long, not only the underlying condition itself, but also disuse of the oral and pharyngeal musculature decompensates swallowing function further.^4^ Therefore, it is important to implement continuous dysphagia rehabilitation to avoid disuse atrophy. In both Japan and Western countries, respiratory infection and failure are the most common causes of death following alternative nutrition methods. In the majority of cases, this is due to pneumonia.^5^, ^6^ Pneumonia in elderly patients is also often caused by tracheal aspiration. Aspirating saliva that may be infected from poor oral care may also lead to pneumonia.^7^ Even if daily oral intake cannot be resumed, dysphagia rehabilitation is important to prevent pneumonia.

As alternative nutrition methods can be anticipated to improve the patient's general nutritional status, continuous dysphagia rehabilitation may improve swallowing function. However, in many cases, once the choice of alternative nutrition method has been made, oral intake proves impossible to resume. One reason for this is that although contraindications for oral intake and indication criteria for enteral and parenteral nutrition have been formulated,^8^ there are no clear indicators for the resumption of oral intake. Although attempts to assess swallowing function and wean patients from feeding tubes following enteral or parenteral nutrition have been reported,^9^, ^10^ these have mostly focused on acute^11^ or convalescent^12^, ^13^ inpatients, and few studies have addressed patients living at home.

The objective of the present study was to consider the effect of dysphagia rehabilitation and investigate parameters associated with the resumption of oral intake in elderly patients receiving home nursing care who were unable to eat by mouth.

## 2 METHODS

### 2.1 Participants

This retrospective cohort study was conducted in a clinic, specialised in rehabilitation of patients with dysphagia, located in Tokyo, Japan.

The study participants were 116 patients (66 men and 50 women, mean age, 79.7 ± 8.9 years). The recruitment period was the 6‐year period from April 2013 to March 2019.

The inclusion criteria were patients who were aged ≥65 years, had requested treatment from our dysphagia rehabilitation clinic, were receiving home nursing care and were taking enteral nutrition because they were incapable of swallowing. The participants of all were not eating by mouth at the initial examination. The exclusion criteria were patients who had progressive neuromuscular diseases (Parkinson disease, spinocerebellar degeneration, amyotrophic lateral sclerosis, myasthenia gravis, progressive supranuclear palsy, corticobasal degeneration and muscular dystrophy) and those lost to follow‐up because of death or hospital admission.

The participants’ demographics at baseline are presented in Table 1. Basic data on the underlying condition causing dysphagia (main disease), main caregiver, type of enteral nutrition, level of consciousness (alertness), duration of enteral nutrition, body mass index, history of pneumonia, ability to walk and swallowing function were obtained at the first home visit. These data served as the baseline measures.

**Table 1:** Participant demographics at baseline

		N (%)
Main disease, n (%)	Cerebrovascular disease	103 (88.8)
	Head injury	7 (6.0)
	Oropharyngeal cancer	6 (5.2)
Main caregiver, n (%)	Husband/wife	69 (59.5)
	Son/daughter	47 (40.5)
Type of enteral nutrition	Percutaneous endoscopic gastrostomy (PEG)	101 (87.1)
	Total parenteral nutrition (TPN)	7 (6.0)
	Nasogastric tube	8 (6.9)
Level of consciousness, n (%)	0 (Lucid)	39 (33.6)
	I	52 (44.8)
	II	25 (21.6)
	III	0 (0)
Duration of enteral nutrition, n (%)	≥6 mo	52 (44.8)
	<6 mo	64 (55.2)
Body mass index, n (%)	≥18.5	41 (35.3)
	<18.5	75 (64.7)
History of pneumonia, n (%)	No	36 (31.0)
	Yes	80 (69.0)
Ability to walk	Able to walk	16 (13.8)
	Able to sit	33 (28.4)
	Unable to walk and sit	67 (57.8)
Swallowing function	Mild	54 (46.6)
	Moderate	15 (12.9)
	Severe	47 (40.5)

Level of consciousness was evaluated by the Japan Coma Scale (JCS).^14^ The JCS is widely used as a scale for evaluating the consciousness level in Japan. The JCS scores are defined as follows^15^: (0), alert consciousness; (I), wakefulness without any stimuli; (II), arousal by some stimuli; and (III), coma. Each category is further classified into three subcategories with code.^16^ JCS and Glasgow Coma Scale assessments are well‐correlated.^17^ The patients were divided into two groups to analyse: the non‐coma group (JCS score: <II) and the coma group (JCS score: ≥II).

Body mass index (BMI) was calculated in the usual manner (weight in kilograms divided by height in metres squared).

Physical function was assessed ability to walk. The categories are consisted as follows: (a) able to walk, (b) able to sit without help and (c) unable to walk and sit without help.

This study was approved by the Institutional Review Board of the Nippon Dental University School of Life Dentistry (NDU‐T2017‐37), and written informed consent was obtained from the patient or his/her family.

### 2.2 Procedure

#### 2.2.1 Assessment of swallowing function

Swallowing function was assessed using the Hyodo score^18^ on flexible endoscopic evaluation of swallowing (FEES) by dysphagia rehabilitation specialists. The Hyodo scoring method is widely used as an objective evaluation for predicting aspiration in Japan. This method consists of four parameters: (a) salivary pooling at the vallecula and piriform sinuses, (b) glottal closure reflex induction by touching the epiglottis or arytenoid with the endoscope, (c) swallowing reflex initiation assessed by “white‐out” timing and (d) pharyngeal clearance after swallowing blue‐dyed water. These four parameters are each scored 0‐3 on a 4‐point scale (0, normal; 1, mildly impaired; 2, moderately impaired; and 3, severely impaired). The Hyodo score is expressed as the sum of scores for each of the four parameters, ranging from 0 to 12.

Patients’ swallowing function was divided into mild (0‐4 points), moderate (5‐8 points) or severe (9‐12 points).^18^

#### 2.2.2 Oral intake status

Oral intake status was assessed using the Functional Oral Intake Scale (FOIS).^19^ FOIS consisted of 7 levels. FOIS 1 means “No oral intake,” FOIS 2 means “Tube‐dependent with minimal/inconsistent oral intake,” FOIS 3 means “Tube supplemented with consistent oral intake,” FOIS 4 means “Total oral intake of a single consistency,” FOIS 5 means “Total oral intake of multiple consistencies requiring special preparation,” FOIS 6 means “Total oral intake with no special preparation, but must avoid specific foods or liquid items,” and FOIS 7 means “Total oral intake with no restrictions.” FOIS 1‐3 are tube‐dependent, and FOIS 4‐7 are total oral intake.

All participants were FOIS 1 on initial examination.

Patients whose FOIS score was 1 after 6 months were classified as being incapable of oral intake, while those whose FOIS score was ≥2 after 6 months were classified as capable of oral intake. Those whose FOIS score was ≥3 were classified as capable of resuming daily oral intake.

#### 2.2.3 Dysphagia rehabilitation

All participants started oral care, oral motor exercises (without using food) and postural adjustments (body posture and head posture) after initial examination. They were instructed by dentists and dental hygienists specialising in swallowing therapy and underwent the exercises by themselves for thirty minutes per day. We checked and had them do the exercises every other week. In addition, based on the assessment of the swallowing function, individual dysphagia rehabilitation was performed in participants’ home once a month for 1 hour at a time. When participants started oral intake, they were assessed on FEES and lectured on how to make modified food and nutritional support^20^, ^21^, ^22^ by a dietitian. All participants continued dysphagia rehabilitation for 6 months with the objective of resuming oral intake. Their oral intake status was evaluated at the end of this period.

We analysed factors associated with starting oral intake and with the resumption of daily oral intake.

### 2.3 Statistical analysis

IBM SPSS statics version 22 for Windows (IBM Japan®) was used for all statistical analyses. Results before and after the 6‐month intervention were compared using a Wilcoxon test. Factors associated with the resumption of oral intake were analysed using a chi‐squared test and logistic regression analysis. The level of statistical significance was set at *P* <.05.

From an ethical perspective, care was taken to protect the participants' personal data, and data management, analysis and testing were performed in a format that did not allow the identification of individuals.

## 3 RESULTS

After initial examination, 38 cases (32.8%) started direct rehabilitation (used modified food) immediately, 42 cases (36.2%) underwent within 6 months, and 36 cases (31.0%) could not start within 6 months. The 36 cases were classified as being incapable of oral intake (FOIS 1).

Oral intake status after 6 months was FOIS 1 in 36 cases (31.0%), FOIS 2 in 50 (43.1%), FOIS 3 in 24 (20.7%), FOIS 4 in 2 (1.7%), FOIS 5 in one (0.9%), FOIS 6 in two (1.7%) and FOIS 7 in one (0.9%), a significant improvement compared with that on initial examination (*P* < .001). Only six patients had completely switched to oral intake (FOIS ≥ 4), with the majority combining oral intake with enteral nutrition.

Table 2 shows the results of our analysis of factors associated with starting oral intake. Significant associations were found with JCS (*P* = .011), ability to walk (*P* = .035) and swallowing function (*P* < .001) by chi‐squared test. In the logistic regression analysis, swallowing function was identified as independent predictive factor. (*P* < .001).

**Table 2:** Analysis of factors associated with starting oral intake

Factor		Starting oral food intake		*P* value
		Able	Unable	
Sex, n	Men	47	19	.548
	Women	33	17	
Age, y	<75	22	10	.975
	≥75	58	26	
History of pneumonia	No	24	12	.720
	Yes	56	24	
Duration of enteral nutrition	≤6 mo	43	21	.646
	>6 mo	37	15	
BMI	≥18.5	28	13	.908
	<18.5	52	23	
JCS	<Ⅱ	68	23	.011
	≥Ⅱ	12	13	
Ability to walk	Able to walk	15	1	.035
	Able to sit	24	9	
	Unable to walk and sit	41	26	
Swallowing function	Mild	51	3	<.001
	Moderate	12	3	
	Severe	30	17	

Abbreviations: BMI, body mass index; JCS, Japan coma scale.

Table 3 shows the results of our analysis of factors associated with the resumption of daily oral intake. Significant associations were found with the JCS (*P* = .021), ability to walk (*P* < .001) and swallowing function (*P* < .001) by chi‐squared test. In the logistic regression analysis, swallowing function (*P* < .001) and ability to walk (*P* < .001) were identified as independent predictive factors.

**Table 3:** Analysis of factors associated with daily oral intake

Factor		Resumption of daily oral food intake		*P* value
		Able	Unable	
Sex, n	Men	20	46	.209
	Women	10	40	
Age, y	<75	8	24	.896
	≥75	22	62	
History of pneumonia	No	9	27	.887
	Yes	21	59	
Duration of enteral nutrition	≤6 mo	19	45	.297
	>6 mo	11	41	
BMI	≥18.5	8	33	.248
	<18.5	22	53	
JCS	<Ⅱ	28	63	.021
	≥Ⅱ	2	23	
Ability to walk	Able to walk	10	6	<.001
	Able to sit	15	18	
	Unable to walk and sit	5	62	
Swallowing function	Mild	22	32	<.001
	Moderate	5	10	
	Severe	3	44	

Abbreviations: BMI, body mass index; JCS, Japan coma scale.

## 4 DISCUSSION

In the present study, even under the restricted circumstances of maintenance‐phase home nursing care, 69.0% of the participants resumed oral intake, and 25.9% were able to resume daily oral intake. The benefits of physical rehabilitation during the maintenance phase include reducing the risk of hospital admission,^23^ improving QOL^23^ and reducing mortality.^24^ Previous studies have also demonstrated that dysphagia rehabilitation may have some effect. In a study by Parmasothy et al^4^ that investigated whether patients were able to resume oral intake at 3 and 6 months after gastrostomy formation, the authors described this as a predictor of ultimately switching to oral intake. However, Mathus‐Vliegen et al^25^ and Naik et al^26^ reported that patients were readmitted to hospital or died before achieving resumption of oral intake, making re‐evaluation difficult. In the present study, the duration of enteral nutrition had no effect on whether oral intake was resumed, indicating that it may be possible to resume oral intake even after having received enteral nutrition for 6 months. Assessment and engagement with a view to the long‐term continuous resumption of oral intake are therefore important. Improving swallowing function is essential to resuming oral intake. Currently, however, even if swallowing function has improved, it is rarely re‐evaluated, and there may be a large pool of individuals who are capable of oral intake but in whom this goes unrecognised. Therefore, it may be important to regularly reassess swallow function in patients who are sent home on enteral nutrition.

As the importance of combining rehabilitation and dietary therapy has been recognised in recent years,^27^ it has become more likely that the nutritional status of patients who have not been eating by mouth may have been improved by enteral nutrition, potentially enabling the resumption of oral intake. In the present study, we attempted to use BMI to investigate the association between nutritional status and whether oral intake was resumed; however, there was no correlation.

We investigated factors associated with starting oral intake and the resumption of daily oral intake. Swallowing function was the most important factor for resuming oral intake. From the viewpoint of aspiration risk, recovery of swallowing function is important for starting oral intake. Resumption of daily oral intake was associated with level of consciousness, ability to walk and swallowing function. The association between level of consciousness and the success or failure of oral intake has previously been reported,^28^ A lower level of consciousness is also associated with the risk of aspiration pneumonia^29^ and is considered to be an important factor for predicting the effectiveness of swallowing rehabilitation training and the resumption of oral intake. Most of these previous studies examined resumption of oral intake in acute or convalescent patients. We obtained similar results from elderly maintenance‐phase patients receiving home nursing care. The maintenance of level of consciousness is related to active training. The ability to maintain body position, adjust the size of mouthfuls and cough after swallowing is important to incorporate oral intake on a daily basis. These factors were noted during dysphagia rehabilitation. The results of the present study suggest that maintaining a sufficient level of consciousness to perform these tasks is required for the resumption of daily oral intake.

Dam et al^30^ and Toh et al^31^ reported that activities of daily living have a major effect on dysphagia rehabilitation treatment in patients with neurological disorders who have received a gastrostomy. In this study, we also found that maintaining the ability to walk was a condition for resuming oral intake. The maintenance of the ability to walk suggests that the patient has the basic muscle strength required for maintaining a seated position which is important for protecting the upper airway while eating.

The limitation of this study was that it only included patients who could be followed for 6 months, and the question of how to deal with dropouts due to hospitalisation or death is an issue for further investigation. We also excluded patients with progressive conditions, such as Parkinson's disease, and other neuromuscular disorders, such as multiple sclerosis. The results may be different for patients with these conditions. Further studies involving larger numbers of patients are required to identify the characteristics of different diseases.

Our results demonstrated the importance of continuous assessment and dysphagia rehabilitation for patients receiving enteral nutrition at home.

## 5 CONCLUSION

We carried out dysphagia rehabilitation with the objective of enabling dysphagia patients who were receiving home nursing care and undergoing enteral nutrition to resume oral intake and found that oral intake function improved after rehabilitation.

Swallowing function was associated with the resumption of oral intake. In addition, anyone who cannot walk may not recover daily oral intake.

## CONFLICT OF INTERESTS

The authors declare no conflict of interest.

## AUTHOR CONTRIBUTIONS

Dr Kikutani had full access to all of the data in the study and took responsibility for the integrity of the data and the accuracy of the data analysis. Furuya, Kikutani and Tamura conceived and designed the study. Furuya, Igarashi, Sagawa, Yajima, Tohara and Takahashi acquired the data. Kikutani, Machida and Tamura analysed and interpreted the data. Kikutani and Tamura drafted the manuscript. Kikutani and Tamura critically revised the manuscript for important intellectual content. Kikutani supervised the study.
